# Full Length Bid is sufficient to induce apoptosis of cultured rat hippocampal neurons

**DOI:** 10.1186/1471-2121-8-7

**Published:** 2007-02-27

**Authors:** Hans-Georg König, Markus Rehm, Daniel Gudorf, Stan Krajewski, Atan Gross, Manus W Ward, Jochen HM Prehn

**Affiliations:** 1 Department of Physiology and RCSI Neuroscience Research Centre, Royal College of Surgeons in Ireland, 123 St Stephen's Green, Dublin 2, Ireland; 2 Interdisciplinary Center for Clinical Research, University Münster Clinics, D-48149 Münster, Germany; 3 The Burnham Institute, Program on Apoptosis and Cell Death, La Jolla, CA 92037, USA; 4 Department of Biological Regulation, Weizmann Institute of Science, Rehovot 76100, Israel

## Abstract

**Background:**

Bcl-2 homology domain (BH) 3-only proteins are pro-apoptotic proteins of the Bcl-2 family that couple stress signals to the mitochondrial cell death pathways. The BH3-only protein Bid can be activated in response to death receptor activation via caspase 8-mediated cleavage into a truncated protein (tBid), which subsequently translocates to mitochondria and induces the release of cytochrome-C. Using a single-cell imaging approach of Bid cleavage and translocation during apoptosis, we have recently demonstrated that, in contrast to death receptor-induced apoptosis, caspase-independent excitotoxic apoptosis involves a translocation of full length Bid (FL-Bid) from the cytosol to mitochondria. We induced a delayed excitotoxic cell death in cultured rat hippocampal neurons by a 5-min exposure to the glutamate receptor agonist N-methyl-D-aspartate (NMDA; 300 μM).

**Results:**

Western blot experiments confirmed a translocation of FL-Bid to the mitochondria during excitotoxic apoptosis that was associated with the release of cytochrome-C from mitochondria. These results were confirmed by immunofluorescence analysis of Bid translocation during excitotoxic cell death using an antibody raised against the amino acids 1–58 of mouse Bid that is not able to detect tBid. Finally, inducible overexpression of FL-Bid or a Bid mutant that can not be cleaved by caspase-8 was sufficient to induce apoptosis in the hippocampal neuron cultures.

**Conclusion:**

Our data suggest that translocation of FL-Bid is sufficient for the activation of mitochondrial cell death pathways in response to glutamate receptor overactivation.

## Background

Excitotoxic neuron death has been implicated in the pathogenesis of ischemic, traumatic, and seizure-induced brain injury [1]. When glutamate receptor overactivation is intense, cell death is necrotic and characterized by a disturbance of cellular ion and volume homeostasis, leading to mitochondrial membrane potential (ΔΨm) depolarization, free radical production, ATP depletion and early plasma membrane leakage [2-5]. However, when glutamate receptor overactivation is subtle, mitochondria transiently recover their energetics, and a delayed cell death may result [3,6,7]. Under these conditions, excitotoxic neuron death is associated with the release of the pro-apoptotic factors cytochrome-C (cyt-C) and Apoptosis-Inducing Factor (AIF) from mitochondria [6-10].

The mechanisms of cyt-C and AIF release during excitotoxic neuron death remain unresolved. In the evolutionary conserved apoptosis pathway, the release of cyt-C requires the pro-apoptotic Bcl-2 family members Bax or Bak [11]. Both proteins are believed to form megachannels in the outer mitochondrial membrane large enough to release intermembrane space proteins [12]. In order to cause this increased permeability, Bax and Bak undergo a conformational change and insert into the outer mitochondrial membrane [13,14]. In non-apoptotic cells, activation of Bax or Bak is inhibited by anti-apoptotic Bcl-2 family members such as Bcl-2 and Bcl-xL. In apoptotic cells, the transcriptional induction or post-translational activation of Bcl-2-homolgy domain-3 (BH3)-only proteins overcomes this inhibition and triggers the activation of Bax and Bak and the release of cyt-C [15,16]. The release of AIF in excitotoxicity and apoptosis is likewise inhibited by Bcl-2 [10,17], suggesting that the activation of BH3-only proteins may also be required to relieve a Bcl-2 inhibition of AIF release.

Previous studies have indicated an involvement of the BH3-only protein Bad in glutamate- and Ca^2+^-induced neuronal apoptosis [18]. Interestingly, neurons from mice deficient in the BH3-only protein Bid have also been shown to be resistant to ischemic injury *in vivo*, as well as hypoxic and excitotoxic injury *in vitro *[19]. Bid is an essential component of most forms of death receptor-mediated apoptosis, and is activated post-translationally via caspase-8-mediated cleavage into a truncated form (tBid) [20,21]. tBid is subsequently myristoylated [22], triggers the activation of Bak or Bax [23,24], and induces cyt-C release from mitochondria. However, there is a growing body of evidence suggesting that caspase activation during excitotoxic neuron death may be marginal or even absent [6,8,25-27]. Conversely, this suggests that Bid may trigger excitotoxic cell death through more than one pathway. Using a fluorescence resonance energy transfer single-cell imaging approach of Bid cleavage and translocation during apoptosis, we have recently demonstrated that caspase-independent excitotoxic apoptosis induces a translocation of full length Bid (FL-Bid) from the cytosol to mitochondria [28]. In the present study, we demonstrate FL-Bid is sufficient to induce apoptosis of cultured rat hippocampal neurons.

## Results

### FL-Bid is not a significant protease substrate during excitotoxic neuron death

**Figure 1:**
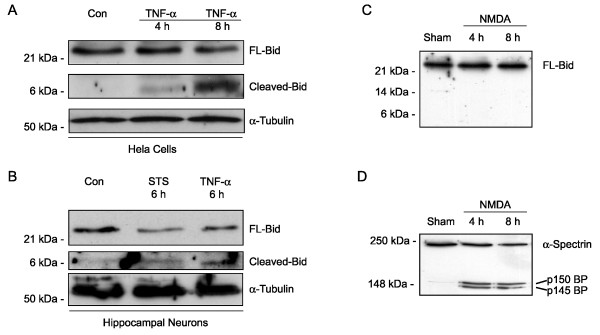
**Death receptor mediated cleavage of Bid in HeLa cells and cultured rat hippocampal neurons**. Cleavage of FL-Bid in HeLa D98 cells (**A**)and cultured rat hippocampal neurons (**B**)exposed to TNF-α plus CHX (100 ng/ml plus 1 μg/ml) or staurosporine (STS, 3 μM and 300 nM, respectively) for the indicated time periods. Bid cleavage was detected by Western blot analysis. Duplicate experiments yielded similar results. (**C**)No detectable cleavage of FL-Bid during NMDA-induced neuronal death. Cultured rat hippocampal neurons were exposed to 300 μM NMDA in Mg^2+^-free HBS for 5 min, washed, returned to the original culture medium, and analyzed by Western blotting after 4 and 8 h. Sham-washed cultures were exposed to Mg^2+^-free HBS devoid of NMDA. Lack of Bid cleavage during excitotoxic neuron death was observed in four separate experiments. (**D**)Immunoblot analysis demonstrates significant cleavage of α-spectrin into its calpain-generated 150 and 145 kDa breakdown products. Duplicate experiments yielded similar results.

To investigate the involvement of apoptotic proteins in excitotoxic neuron death, we recently established a model in which a transient, 5-min exposure to the glutamate receptor agonist NMDA (300 μM) induced a delayed cell death in primary cultures of rat hippocampal neurons [7,8]. This excitotoxic cell death is characterized by mitochondrial cyt-C release and ΔΨm depolarization setting in 4 to 8 h after the NMDA exposure, followed by nuclear condensation and cell shrinkage [7,8]. We determined if FL-Bid is able to translocate to mitochondria in this model of excitotoxic apoptosis. To this end, immunoblotting experiments were performed using a rabbit polyclonal antibody (AR-53) that detects p21 FL-Bid, as well as its caspase-8-/caspase-3-generated NH_2_-terminal cleavage product [29,30]. Control experiments demonstrated that the antibody was able to detect FL-Bid and its caspase-generated NH_2_-terminal p7 fragment in HeLa D98 cells after an activation of death receptors with TNF-α/Cycloheximide(CHX) (Fig. 1A, see also Additional File 1A). The antibody was also able to detect the p7 fragment in cultured rat hippocampal neurons (Fig. 1B) exposed to the apoptosis-inducing kinase inhibitor staurosporine (STS) or TNF-α/CHX (Fig. 1B see also Additional File 1B). However, neither a decrease in the content of FL-Bid, nor an accumulation of caspase-generated cleavage products could be detected in whole cell lysates of cultured rat hippocampal neurons exposed to NMDA (Fig. 1C, see also Additional File 1C). Activation of proteases after the NMDA exposure was however clearly detectable in the same whole cell lysates. This was evident from the decrease in the calpain I substrate full-length α-spectrin and the accumulation of calpain-specific 150 and 145 kDa α-spectrin cleavage products 4 and 8 h after the NMDA exposure (Fig. 1D).

### Translocation of FL-Bid to mitochondria during excitotoxic neuron death

**Figure 2:**
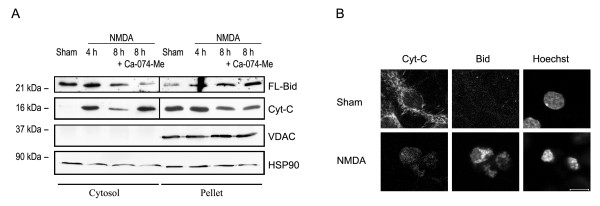
**Translocation of FL-Bid to the mitochondria coincides with a loss of cyt-C**. (**A**)Immunoblot analysis of cytosolic and mitochondria-containing pellet fractions after selective plasma membrane permeabilization in cells exposed to Mg^2+^-free HBS (controls) or NMDA with or without CA-074-Me. The experiment was performed in duplicate with similar results. (**B**)Immunofluorescence analysis of cyt-C and Bid distribution in sham- and NMDA-exposed rat hippocampal neurons. Cells were fixed 8 h after termination of the exposure. Nuclei were counterstained with Hoechst 33258. Scale bar = 5 μm.

Next, we performed selective plasma membrane permeabilization and subsequent immunoblotting of the cytosolic fraction and the mitochondria-containing pellet fraction in cultured rat hippocampal neurons exposed to NMDA for 5 min. Immunoblotting with a voltage dependent anion carrier (VDAC) antibody demonstrated that the cytosolic fraction was not contaminated with mitochondria. Immunoblotting with the Bid antibody revealed that p21 FL-Bid translocated from the cytosol to the mitochondria-containing pellet fraction 4 h and more pronounced 8 h after termination of the NMDA exposure (Fig. 2A). This process was paralleled by the translocation of cyt-C from the mitochondria-containing pellet fraction to the cytosol (Fig. 2A) 4 h and 8 h after the NMDA exposure. Interestingly, despite a significant cyt-C decrease in the mitochondrial fraction by 8 h, the cyt-C content in the cytosolic fraction did not increase correspondingly, suggesting that cyt-C may be degraded upon release into the cytosol (see also [31]). Indeed, treatment with the membrane permeable cathepsin inhibitor CA-074 methyl ester recovered the cyt-C content in the cytosolic fraction 8 h after the NMDA exposure (Fig. 2A).

These results were confirmed by immunofluorescence analysis of Bid redistribution during excitotoxic neuron death using the above described Bid antibody. Neurons of sham-exposed control cultures exhibited a diffuse Bid immunofluorescence signal (Fig. 2B). Cyt-C co-staining revealed a filamentous, punctate staining pattern in sham-exposed controls characteristic of mitochondria. In contrast, cells that had released cyt-C in response to NMDA exhibited a clustered Bid immunofluorescence around the nucleus. Cells with released cyt C and clustered Bid immunofluorescence also exhibited nuclear chromatin condensation as evidenced by staining with the chromatin dye Hoechst 33258 (Fig. 2B).

### Mild overexpression of FL-Bid or a Bid mutant that can not be cleaved by caspase-8 potently induces cell death in cultured rat hippocampal neurons

**Figure 3:**
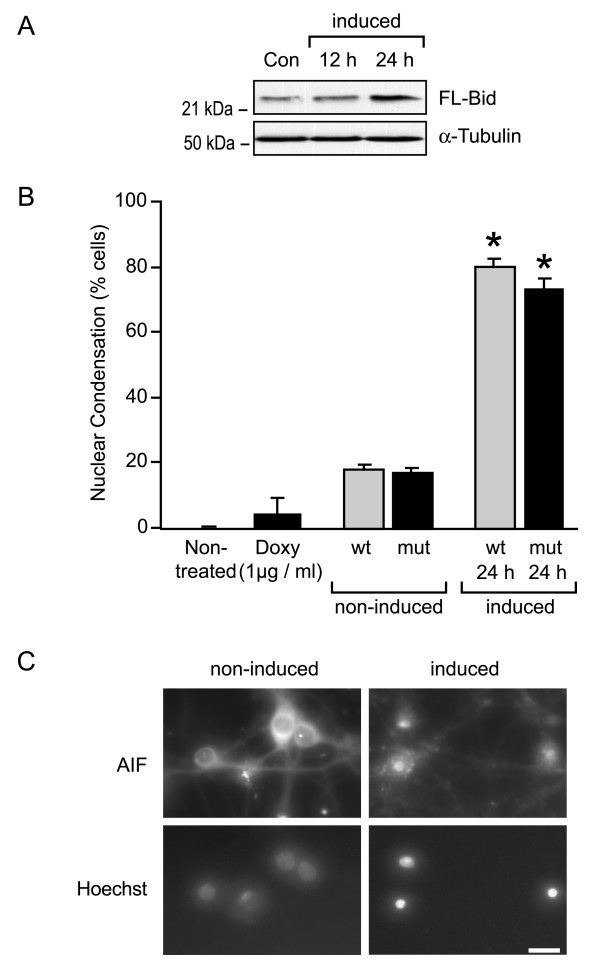
**FL-Bid or a Bid mutant that can not be cleaved by caspase-8 potently induces cell death in cultured rat hippocampal neurons**. (**A**)Western blot analysis of Bid overexpression in cultured rat hippocampal neurons. Cells were co-infected with the wild-type (wt) FL-Bid adenovirus and the rtTA containing virus. Expression of Bid was induced by 1 μg/ml doxycycline treatment for 12 or 24 h. Control cells were infected but not induced. (**B**)Quantification of cells showing nuclear condensation in response to an overexpression of wt FL-Bid or the Bid(D59A) mutant (mt). Cells were co-infected with the wt or mt FL-Bid adenovirus and the rtTA containing virus. After 48 h, expression of Bid was induced by the addition of 1 μM doxycycline for 12 or 24 h. Cells exhibiting nuclear condensation or nuclear fragmentation were counted in 4 – 5 randomly chosen subfields after staining with the chromatin-specific dye Hoechst 33528. Data are means ± SEM from n = 3 experiments. (**C**)AIF immunofluorescence analysis in non-induced and induced hippocampal neurons. Cells were co-infected with the wt FL-Bid adenovirus and the rtTA containing virus and induced for 12 h. Note the mitochondrial AIF immunofluorescence in non-induced cells sparing the nuclear region, and AIF translocation to the nucleus in the induced neuron cultures. Bar = 10 μm.

We subsequently addressed the question, whether FL-Bid was sufficient to induce cell death in the hippocampal neuron cultures, and whether this cell death can occur in the absence of caspase-8-mediated Bid cleavage. Cultured rat hippocampal neurons were infected with adenoviral vectors expressing either FL-Bid or a Bid mutant that can not be cleaved by caspase-8 (D59A) under the control of a tetracycline responsive promoter [32]. Western blot analysis of cultures infected with the adenoviral vectors (50 MOI) and induced for 24 h with 1 μg/ml doxycycline demonstrated a mild overexpression of FL-Bid in the hippocampal neuron cultures (Fig. 3A). This overexpression was however sufficient to induce a massive cell death in the hippocampal neuron cultures that was characterized by cell shrinkage and nuclear condensation. Hoechst staining of nuclear chromatin revealed significant neuronal damage 24 h after induction with doxycycline, reaching a level of 80.1 ± 2.7% (Fig. 3B). FL-Bid-induced cell death was associated with the mitochondrial release of the pro-apoptotic factors cyt-C and AIF (Fig. 3C and data not shown). In contrast, hippocampal neurons that were infected with the adenoviral vectors but were not induced with doxycycline remained viable for up to 24 h, as were cells that were treated with doxycycline but not infected with the adenoviral vectors (Fig. 3B). Interestingly, overexpression of Bid(D59A) also potently induced cell death in the hippocampal neuron cultures, reaching a level of 73.5 ± 3.1% after 24 h (p > 0.1, no significant difference compared to FL-Bid induced cultures).

## Discussion

Ischemic and hypoxic injuries to the nervous system have been shown to involve the release of cell death-inducing cytokines and the activation of death receptors [19]. It is likely that these events involve the caspase-8-mediated cleavage of the BH3-only protein Bid. In support of this hypothesis, Bid-deficient animals exhibited reduced neuronal injury after cerebral ischaemia [19]. Our data provide evidence that Bid may be involved in an additional, intrinsic cell death pathway triggered by the overactivation of glutamate receptors. This pathway did not require the generation of the caspase-8-generated cleavage product tBid. Instead, we observed an efficient translocation of FL-Bid to mitochondria during excitotoxic neuron death. This translocation was associated with the mitochondrial release of pro-apoptotic factors, a process that commits cells to both caspase-dependent and caspase-independent cell death [33,34]. Although our data does not rule out the possibility that other BH3 only proteins are involved in excitotoxic apoptosis, it demonstrates that FL-Bid is sufficient to induce an apoptotic cell death in cultured rat hippocampal neurons.

There is growing evidence that the caspase cascade may not be potently activated during excitotoxic neuron death, despite the release of cyt-C from mitochondria [6-10,27,28]. We and others have previously demonstrated that calpains activated during excitotoxicity degrade and inactivate essential components of the caspase activating pathway including APAF-1, procaspase-9, -8, and -3 [8,35,36]. There is also evidence that levels of Apaf-1 decrease during neuronal maturation, inhibiting apoptosome formation after mitochondrial outer membrane permeabilization (MOMP) [37]. In the absence of caspase activation MOMP may activate alternative cell death pathways that include ATP depletion and increased ROS production subsequent to cyt-C release, as well as the release of AIF [7,9,38-40].

In contrast to death receptor-induced apoptosis we could not detect significant amounts of cleaved Bid accumulating in the cultured rat hippocampal neurons in response to NMDA. However, we cannot exclude the possibility that Bid may be cleaved during the later stages of apoptosis, downstream of MOMP or an intracellular ion homeostasis breakdown [28,41,42]. These cells will subsequently undergo plasma membrane leakage, a process that may limit the ability to detect Bid cleavage during the late stages of apoptosis. A previous report has demonstrated that translocation of FL-Bid to mitochondria may also occur in response to an activation of death receptors when caspase-8 activation is blocked by the addition of a caspase-8 inhibitor [43]. In Bid- and caspase-8-deficient mouse embryonic fibroblasts, FL-Bid or the non-cleavable mutant FL-Bid(D59A) are also sufficient to activate apoptosis [32]. In line with this finding we found no significant difference in the ability of FL-Bid and Bid(D59A) to induce cell death in cultured rat hippocampal neurons.

How may NMDA receptor overactivation stimulate FL-Bid translocation? Previous studies have demonstrated that excitotoxic neuron death is associated with a selective inhibition of phosphatidylcholine synthesis [44]. It has also been shown that physiological concentrations of phosphatidic acid and phosphatidylgycerol are able to induce an accumulation of FL-Bid in mitochondria [45]. FL-Bid has been shown to be sufficient to induce the oligomerization of Bax/Bak, resulting in its integration into the outer mitochondrial membrane triggering cyt-C release [24]. Finally, recombinant FL-Bid displayed lipid transfer activity under the same conditions and at the same nanomolar concentrations that lead to mitochondrial translocation and Cyt-C release [45]. Changes in the intracellular phospholipid environment during excitotoxic cell death signals may hence induce the translocation of FL-Bid to mitochondria and may initiate the release of pro-apoptotic factors from mitochondria.

## Conclusion

Bid is highly expressed in the nervous system during embryonic and postnatal development. Interestingly and in contrast to most BH3 only proteins, Bid expression remains high in the adult nervous system [29]. The ability of both tBid and FL-Bid to translocate to mitochondria and to induce cell death suggest that this BH3-only protein is a central mediator of pathophysiological neuron death.

## Methods

### Materials

N-Methyl-D-aspartate (NMDA), recombinant tumor necrosis factor-α (TNF-α), glycin and cycloheximide (CHX) came from Sigma (Poole, Dorset, U.K.). Tetrodotoxin was purchased from Biotrend (Cologne, Germany), CA-074 methyl ester (CA-074-ME) from Calbiochem (Bad Soden, Germany), and staurosporine (STS) from Alexis (Grünberg, Germany).

### Cell Culture

Cultured hippocampal neurons were prepared from neonatal (P1) Fischer 344 rats (Luetjens et al., 2000). Dissociated hippocampal neurons were plated at a density of 2 × 10^5 ^cells/cm^2 ^into poly-L-lysine-coated 6- or 24-well plates or glass chamber slides (Nunc). Cells were maintained in MEM medium supplemented with 10% NU-serum, 2% B-27 supplement (50 × concentrate), 2 mM L-glutamine, 20 mM D-glucose, 26.2 mM sodium bicarbonate, 100 U/ml penicillin and 100 μg/ml streptomycin (Life Technologies, Karlsruhe, Germany). Experiments were performed on 14 – 16 day-old cultures. Animal care followed official governmental guide lines. HeLa D98 cells were cultured in RPMI 1640 medium (Life Technologies, Germany) supplemented with 10% fetal calf serum (PAA, Cölbe, Germany).

### Excitotoxic neuronal injury

Cultures were exposed for 5 min to Mg^2+^-free Hepes-buffered saline (HBS) supplemented with 300 μM NMDA, 0.5 μM tetrodotoxin and 1 μM glycine [7,8]. Control cultures were exposed to Mg^2+^-free HBS devoid of NMDA (sham exposure). Cell death was determined after 24 h by trypan-blue uptake [7]. Experiments were performed on 14 or 15 day-old cultures.

### Generation of adenoviral vectors expressing FL-Bid or FL-Bid(D59A) in an inducible system and infection protocol

Tetracycline (tet)-on inducible adenovirus vectors expressing wild-type FL-Bid or the D59A mutant of FL-Bid that can not be cleaved by caspase-8 were generated as described previously [32]. Hippocampal neurons were infected at a MOI (multiplicity of infection) of 50 with both the FL-Bid or Bid(D59A) containing viruses and the reverse tet transactivator rtTA containing virus. One μg/ml doxycycline (Sigma) was added to the medium 24 h post infection to activate gene expression from the tet-inducible promoter [32].

### Immunofluorescence microscopy

Hippocampal neurons were washed, fixed with formaldehyde, and permeabilized with Tween-20 (0.3%). The following primary antibodies were used: mouse monoclonal anti-cyt-C (6H2.B4, San Diego, CA; 10 μg/ml), rabbit polyclonal anti-Bid raised against amino acids 1–58 of mouse Bid (AR-53; 1:1,000), and rabbit polyclonal anti-Apoptosis-Inducing-Factor (AIF) (1:500; [46]). Nuclei were counterstained with Hoechst33258. Primary antibodies were detected and fluorescent images acquired as described previously [7].

### Digitonization, SDS-PAGE, and Western blotting

The release of cyt-C from mitochondria was analyzed by selective permeabilization of the plasma membrane [47]. Briefly, cells were permeabilized with 100 μg/ml digitonin at 4°C for 10 min. The supernatant representing the cytosol and the mitochondria-containing pellet fraction were separated by centrifugation and denatured. SDS-PAGE and Western blot analysis was performed as described previously [8]. Cyt-C was detected with a monoclonal Cyt-C antibody (7H8.2C12; Pharmingen), Bid with the rabbit polyclonal antibody diluted 1:2,000, VDAC (Porin) and HSP-90 with mouse monoclonal anti-Porin (31HL, Calbiochem) and anti-HSP90α/β (Santa Cruz, Heidelberg, Germany) antibodies, respectively, at a dilution of 1:5,000. α-spectrin and its calpain-generated cleavage products were detected with a mouse monoclonal antibody (1622; Chemicon) diluted 1:5,000, and α-tubulin with a mouse monoclonal antibody (DM-1A; Sigma) diluted 1:1,000.

### Statistics

Data are given as means ± S.E.M. For statistical comparison, one-way analysis of variance followed by Tukey's test were employed. P values smaller than 0.05 were considered to be statistically significant.

## Authors' contributions

HGK carried out the molecular analyses, participated in the sequence alignment and drafted the manuscript.

MR carried out the immunoassays.

DG participated in the sequence alignment.

SK participated in the design of the study and performed the statistical analysis.

MW carried out immunofluorescence, participated in its design and coordination and helped to draft the manuscript.

JHMP conceived of the study, and participated in its design and coordination and helped to draft the manuscript.

All authors have read and approved the final manuscript

## Supplementary Material

Additional File 1 **Long exposures for **Figure 1A, B and 1C. The abundance of the FL-Bid in relation to the cleaved-Bid fragments is depicted on one blot. In contrast to STS and TNF-α treated HeLa cells no such band is visible after treatment of the hippocampal neurons with NMDA. Click here for file
