# Supplementary figures and images for: Full Length Bid is sufficient to induce apoptosis of cultured rat hippocampal neurons
Source: BMC Cell Biol. 2007 Feb 27;8:7. doi: 10.1186/1471-2121-8-7 (PMC1808451; doi:10.1186/1471-2121-8-7)

**Figure 1 A: long exposure**

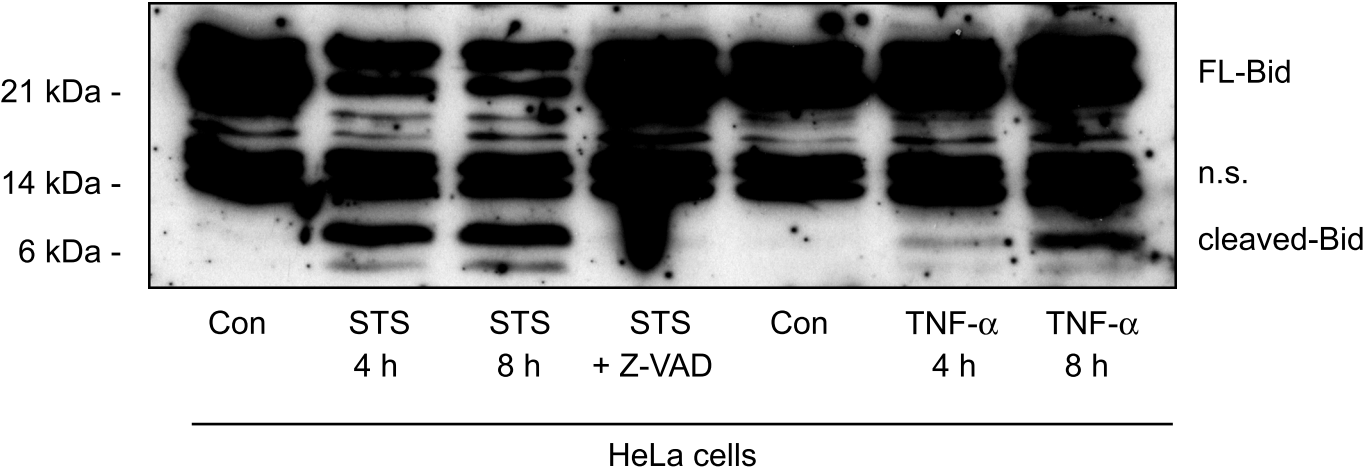

**Figure 1 B: long exposure**

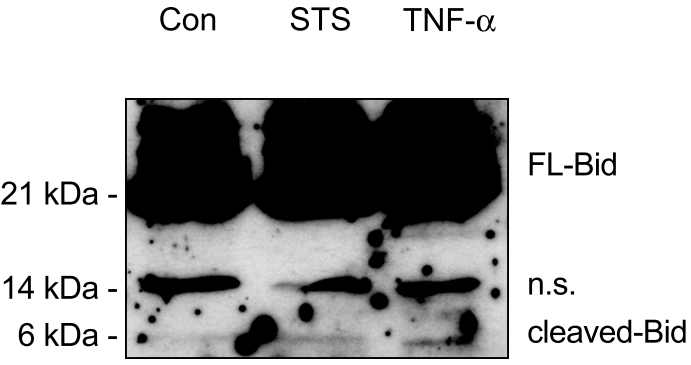

**Figure 1 C: long exposure**

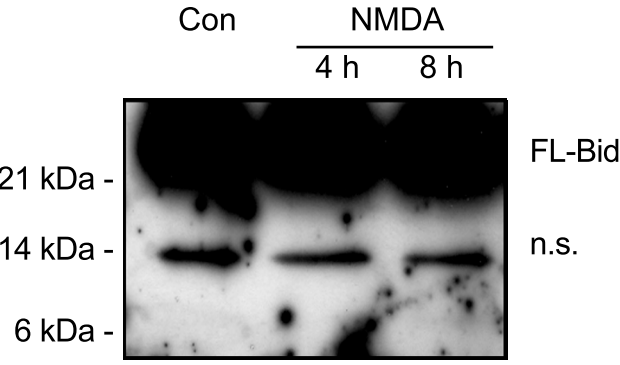

Supplement: Additional File 1 — Long exposures for Figure 1A, B and 1C. The abundance of the FL-Bid in relation to the cleaved-Bid fragments is depicted on one blot. In contrast to STS and TNF-α treated HeLa cells no such band is visible after treatment of the hippocampal neurons with NMDA. [file 1471-2121-8-7-S1.pdf]
